# The Analysis of Teaching of Medical Schools (*AToMS*) survey: an analysis of 47,258 timetabled teaching events in 25 UK medical schools relating to timing, duration, teaching formats, teaching content, and problem-based learning

**DOI:** 10.1186/s12916-020-01571-4

**Published:** 2020-05-14

**Authors:** Oliver Patrick Devine, Andrew Christopher Harborne, Hugo Layard Horsfall, Tobin Joseph, Tess Marshall-Andon, Ryan Samuels, Joshua William Kearsley, Nadine Abbas, Hassan Baig, Joseph Beecham, Natasha Benons, Charlie Caird, Ryan Clark, Thomas Cope, James Coultas, Luke Debenham, Sarah Douglas, Jack Eldridge, Thomas Hughes-Gooding, Agnieszka Jakubowska, Oliver Jones, Eve Lancaster, Calum MacMillan, Ross McAllister, Wassim Merzougui, Ben Phillips, Simon Phillips, Omar Risk, Adam Sage, Aisha Sooltangos, Robert Spencer, Roxanne Tajbakhsh, Oluseyi Adesalu, Ivan Aganin, Ammar Ahmed, Katherine Aiken, Alimatu-Sadia Akeredolu, Ibrahim Alam, Aamna Ali, Richard Anderson, Jia Jun Ang, Fady Sameh Anis, Sonam Aojula, Catherine Arthur, Alena Ashby, Ahmed Ashraf, Emma Aspinall, Mark Awad, Abdul-Muiz Azri Yahaya, Shreya Badhrinarayanan, Soham Bandyopadhyay, Sam Barnes, Daisy Bassey-Duke, Charlotte Boreham, Rebecca Braine, Joseph Brandreth, Zoe Carrington, Zoe Cashin, Shaunak Chatterjee, Mehar Chawla, Chung Shen Chean, Chris Clements, Richard Clough, Jessica Coulthurst, Liam Curry, Vinnie Christine Daniels, Simon Davies, Rebecca Davis, Hanelie De Waal, Nasreen Desai, Hannah Douglas, James Druce, Lady-Namera Ejamike, Meron Esere, Alex Eyre, Ibrahim Talal Fazmin, Sophia Fitzgerald-Smith, Verity Ford, Sarah Freeston, Katherine Garnett, Whitney General, Helen Gilbert, Zein Gowie, Ciaran Grafton-Clarke, Keshni Gudka, Leher Gumber, Rishi Gupta, Chris Harlow, Amy Harrington, Adele Heaney, Wing Hang Serene Ho, Lucy Holloway, Christina Hood, Eleanor Houghton, Saba Houshangi, Emma Howard, Benjamin Human, Harriet Hunter, Ifrah Hussain, Sami Hussain, Richard Thomas Jackson-Taylor, Bronwen Jacob-Ramsdale, Ryan Janjuha, Saleh Jawad, Muzzamil Jelani, David Johnston, Mike Jones, Sadhana Kalidindi, Savraj Kalsi, Asanish Kalyanasundaram, Anna Kane, Sahaj Kaur, Othman Khaled Al-Othman, Qaisar Khan, Sajan Khullar, Priscilla Kirkland, Hannah Lawrence-Smith, Charlotte Leeson, Julius Elisabeth Richard Lenaerts, Kerry Long, Simon Lubbock, Jamie Mac Donald Burrell, Rachel Maguire, Praveen Mahendran, Saad Majeed, Prabhjot Singh Malhotra, Vinay Mandagere, Angelos Mantelakis, Sophie McGovern, Anjola Mosuro, Adam Moxley, Sophie Mustoe, Sam Myers, Kiran Nadeem, Reza Nasseri, Tom Newman, Richard Nzewi, Rosalie Ogborne, Joyce Omatseye, Sophie Paddock, James Parkin, Mohit Patel, Sohini Pawar, Stuart Pearce, Samuel Penrice, Julian Purdy, Raisa Ramjan, Ratan Randhawa, Usman Rasul, Elliot Raymond-Taggert, Rebecca Razey, Carmel Razzaghi, Eimear Reel, Elliot John Revell, Joanna Rigbye, Oloruntobi Rotimi, Abdelrahman Said, Emma Sanders, Pranoy Sangal, Nora Sangvik Grandal, Aadam Shah, Rahul Atul Shah, Oliver Shotton, Daniel Sims, Katie Smart, Martha Amy Smith, Nick Smith, Aninditya Salma Sopian, Matthew South, Jessica Speller, Tom J. Syer, Ngan Hong Ta, Daniel Tadross, Benjamin Thompson, Jess Trevett, Matthew Tyler, Roshan Ullah, Mrudula Utukuri, Shree Vadera, Harriet Van Den Tooren, Sara Venturini, Aradhya Vijayakumar, Melanie Vine, Zoe Wellbelove, Liora Wittner, Geoffrey Hong Kiat Yong, Farris Ziyada, I. C. McManus

**Affiliations:** 1 UCL Medical School, 74 Huntley Street, London, WC1E 6BT UK; 2 Good Hope Hospital, Rectory Rd, Sutton Coldfield, B75 7RR UK; 3 St George’s, University of London, Cranmer Terrace, London, SW17 0RE UK; 4 School of Clinical Medicine, University of Cambridge, Addenbrooke’s Hospital, Hills Rd, Cambridge, CB2 0SP UK; 5 Medical Student Office, Newcastle University, Framlington Place, Newcastle upon Tyne, NE2 4HH UK; 6 Hull University Teaching Hospitals, Hull Royal Infirmary, Anlaby Road, Hull, HU3 2JZ UK; 7 Faculty of Medicine, University of Southampton, Building 85, Life Sciences Building, Highfield Campus, Southampton, SO17 1BJ UK; 8 University of Aberdeen, Suttie Centre, Foresterhill, Aberdeen, AB25 2ZD UK; 9 Norwich Medical School, Faculty of Medicine and Health Sciences, University of East Anglia, Norwich, NR4 7TJ UK; 10 Faculty of Health Sciences, University of Bristol Medical School, First Floor South, Senate House, Tyndall Avenue, Bristol, BS8 1TH UK; 11 Imperial College School of Medicine, South Kensington Campus, London, SW7 2AZ UK; 12 School of Medicine, Dentistry and Nursing, University of Glasgow, Glasgow, G12 8QQ UK; 13 John Hughlings Jackson Building, University of York, Heslington, York, YO10 5DD UK; 14 School of Medicine, Keele University, David Weatherall Building, Keele University Campus, Staffordshire, ST5 5BG UK; 15 Birmingham Medical School, Vincent Drive, Edgbaston, Birmingham, West Midlands B15 2TT UK; 16 University of Edinburgh Medical School, 47 Little France Cres, Edinburgh, EH16 4TJ UK; 17 Brighton and Sussex Medical School, BSMS Teaching Building, University of Sussex, Brighton, BN1 9PX UK; 18 The Medical School, The University of Sheffield, Beech Hill Road, Sheffield, S10 2RX UK; 19 Barts and The London Medical School, 4 Newark St, Whitechapel, London, E1 2AT UK; 20 Cambridge University Hospitals NHS Foundation Trust, Hills Road, Cambridge, CB2 0QQ UK; 21 University of Dundee School of Medicine, 4 Kirsty Semple Way, Dundee, DD2 4BF UK; 22 The University of Nottingham, Queen’s Medical Centre, Nottingham, NG7 2UH UK; 23 Whiston Hospital, Warrington Road, Prescot, L35 5DR UK; 24 Medical Sciences Divisional Office, University of Oxford, Level 3, John Radcliffe Hospital, Oxford, OX3 9DU UK; 25 Guy’s, King’s and St Thomas’ School of Medical Education, Henriette Raphael Building, Guy’s Campus, London, SE1 1UL UK; 26 Queen’s University Belfast, University Road, Belfast, BT7 1NN UK; 27 Manchester Medical School, Stopford Building, Oxford Rd, Manchester, M13 9PT UK; 28 Cardiff University School of Medicine, Cochrane Building, Heath Park Way, Cardiff, CF14 4YU UK; 29 School of Medicine, Worsley Building, University of Leeds, Leeds, LS2 9NL UK; 30 University of Liverpool Medical School, Cedar House, Ashton St, Liverpool, L69 3GE UK; 31 George Davies Centre, University of Leicester School of Medicine, Lancaster Road, Leicester, LE1 7HA UK; 32 St James’s University Hospital, Beckett Street, Leeds, West Yorkshire LS9 7TF UK; 33 Homerton University Hospital, Homerton Row, London, E9 6SR UK; 34 University Hospitals of Leicester NHS Trust, Infirmary Square, Leicester, LE1 5WW UK; 35 Nottingham University Hospitals NHS Trust, Hucknall Rd, Nottingham, NG5 1PB UK; 36 Aberdeen Royal Infirmary, Foresterhill, Aberdeen, AB25 2ZN UK; 37 Research Department of Medical Education, UCL Medical School, Gower Street, London, WC1E 6BT UK

**Keywords:** Medical school differences, Teaching styles, Problem-based learning, Timetables, Lectures, Tutorials, Clinical teaching, Self-regulated learning

## Abstract

**Background:**

*What subjects* UK medical schools teach, *what ways* they teach subjects, and *how much* they teach those subjects is unclear. Whether teaching differences *matter* is a separate, important question. This study provides a detailed picture of timetabled undergraduate teaching activity at 25 UK medical schools, particularly in relation to problem-based learning (PBL).

**Method:**

The Analysis of Teaching of Medical Schools (*AToMS*) survey used detailed timetables provided by 25 schools with standard 5-year courses. Timetabled teaching events were coded in terms of course year, duration, teaching format, and teaching content. Ten schools used PBL. Teaching times from timetables were validated against two other studies that had assessed GP teaching and lecture, seminar, and tutorial times.

**Results:**

A total of 47,258 timetabled teaching events in the academic year 2014/2015 were analysed, including SSCs (student-selected components) and elective studies. A typical UK medical student receives 3960 timetabled hours of teaching during their 5-year course. There was a clear difference between the initial 2 years which mostly contained basic medical science content and the later 3 years which mostly consisted of clinical teaching, although some clinical teaching occurs in the first 2 years. Medical schools differed in duration, format, and content of teaching. Two main factors underlay most of the variation between schools, *Traditional* vs *PBL teaching* and *Structured* vs *Unstructured teaching.* A curriculum map comparing medical schools was constructed using those factors. PBL schools differed on a number of measures, having more PBL teaching time, fewer lectures, more GP teaching, less surgery, less formal teaching of basic science, and more sessions with unspecified content.

**Discussion:**

UK medical schools differ in both format and content of teaching. PBL and non-PBL schools clearly differ, albeit with substantial variation within groups, and overlap in the middle. The important question of whether differences in teaching *matter* in terms of outcomes is analysed in a companion study (*MedDifs*) which examines how teaching differences relate to university infrastructure, entry requirements, student perceptions, and outcomes in Foundation Programme and postgraduate training.

## Background

Medical schools teach. That much is obvious. But *what subjects* they teach, *what ways* they teach subjects, and *how much* they teach each subject in those different ways is very unclear. Harder still is to know whether medical school differences in teaching actually matter. Does greater or lesser *duration* of teaching, in different *formats*, and of different *contents*, produce doctors who perform and practise differently? In this paper, we report the findings of the *AToMs* study which provides empirical answers to the questions of what teaching actually occurs in UK medical schools and how schools differ in their teaching. In a companion paper reporting the *MedDifs* study [1], we describe how differences in teaching format and content relate to a range of different outcome measures. These measures include performance and perceptions during the medical course and afterwards in clinical practice, and how they relate to input measures such as curricular differences, selection processes, and institutional histories.

Recent discourse in medical education, driven particularly by shortages of general practitioners (GPs) and psychiatrists, assumes that differences in teaching result in differences in outcomes. Professor Ian Cumming, the chief executive of Health Education England (HEE), put it straightforwardly when in July 2017 he was quoted as saying:

‘It’s not rocket science. If the curriculum is steeped in teaching of mental health and general practice you get a much higher percentage of graduates who work in that area in future.’ [2]

The UK Royal College of Psychiatrists similarly suggested in October 2017 that:

‘medical schools must do more to put mental health at the heart of the curriculum … and [thereby] encourage more medical students to consider specialising in psychiatry’ [3],

although the President of the College of Psychiatrists did acknowledge that:

‘the data we currently have to show how well a medical school is performing in terms of producing psychiatrists is limited’ [3]

At the heart of that limitation is a lack of detailed quantitative *evidence* on differences in medical school teaching, and only with such data will a proper analysis be possible of the *effects* of medical school differences in teaching. The central aim of this study is to provide such evidence.

Information on teaching carried out by medical schools might be thought to be already available. Certainly, medicine is potentially in a stronger position to know, compared to other university disciplines. The General Medical Council (GMC) acts as the regulator in the UK for undergraduate education, visiting all UK medical schools in a regular cycle. Such reports, though, consist almost entirely of discursive, textual assessments [4]. A detailed comparison between schools is therefore not possible. Other UK bodies such as the Quality Assurance Agency for Higher Education (QAA) have assessed teaching in all university departments, including medical schools in their Teaching Quality Assessments (TQAs). The TQAs were last attempted for medicine in 1998–2000, carried out separately on behalf of the four regional Higher Education Funding Councils for England and Northern Ireland, Scotland, and Wales, with some differences in methodology [5, 6]. The medical schools of England and Northern Ireland were assessed on a scale of 1 to 4 in each of six domains, integrated across the entire medical school curriculum [7]. Recent attempts to create a UK Teaching Excellence Framework (TEF) have so far only provided global assessments at the level of entire universities and provide neither information on medical schools nor details of actual teaching [8]. It should be emphasised that TQA and TEF primarily assess quality rather than content. Finally, some schools such as Manchester [9, 10] have mapped broad content areas of teaching in each year of study, using objectives aligned to the GMC’s *Tomorrow’s Doctors*, and the European Tuning Tags/Medine2 codes [11, 12]. While such maps delineate the intended material to be taught in each year, they do not indicate the specifics of how that teaching takes place and its quantity.

Outside of medicine, a recent and rare attempt to look in detail at teaching within a university discipline is in economics. The innovative *Rethinking Economics* group was set up by economics students in the wake of the financial crisis of 2007–2008 to critique the teaching actually taking place in economic faculties [13]. Universities were asked to participate in a detailed survey, but only seven agreed to do so, with 174 modules being analysed, based on module course outlines and examination papers for the year 2014/2015 [14].

A final source of information about university teaching is the Student Academic Experience Surveys carried out by the Higher Education Policy Institute (HEPI), which is an independent think tank based in Oxford, UK. In 2006, 2007, 2009, and 2012–2017, HEPI carried out large-scale representative surveys of 126,000 students across the UK higher education sector, 5000 of whom were medical students. Most perceptions of teaching are global and generic, but an important feature of the HEPI studies is that students themselves, from named institutions and courses, are asked to provide detailed information on total contact hours for specific formats of teaching.

The few previous studies have taken as units of analysis either *module descriptions* and examination papers (as for economics, with a content analysis used on the texts), or individual students and their *integrated perceptions* (as in the HEPI analyses of contact hours). A different approach uses curriculum maps based primarily on learning objectives, as with the maps produced by the University of Manchester [9], which are not, to our knowledge, available for comparison with other UK medical schools. This study takes a different approach, using *medical school timetables* as the primary sampling frame, with the basic unit of analysis being *timetabled teaching events*, defined as the minimal timed units on a timetable.

### The historical context of medical school teaching and the rise of problem-based learning

Historically, medical curricula in the UK were remarkably constant in their form from the nineteenth century onwards, and then, as Leinster has put it, despite,

‘medical education [being] a very conservative part of a conservative profession, [ … ] in the early 1990s change swept through UK medical schools [as] medical school curricula, which had been relatively homogenous, became diverse in terms of teaching methods and contents … ’ [15](p. 1).

Change was driven by several forces. The GMC had tried unsuccessfully to alter teaching in the *Recommendations* it published in 1947, 1957, and 1980 [16]. That changed with the GMC’s *Tomorrow’s Doctors* [17] of 1993 which gave official support to innovation, with proposals that factual overload in traditional curricula should be reduced by a slimmed down core curriculum, supplemented by special study modules (now student-selected components (SSCs)), comprising perhaps one third of teaching, for developing intellectual skills, curiosity, and critical thinking. The major educational innovation for the new and revised courses was mostly the use of problem-based learning (PBL) courses, a method developed half a century ago, at McMaster, Maastricht, and Harvard [18–20]. As with many educational approaches, PBL is not a rigid and fixed approach to a curriculum, but instead, there is ‘great variability’ [21, 22], with many species and subspecies [23]. A recent review suggested that PBL should be regarded as a toolbox of techniques, including, for instance, case-based learning [21]. The newer medical curricula contain a range of different approaches, including ‘end [ing] … the division between pre-clinical and clinical years, … earlier contact with patients and greater interactions with teachers’ [24] (p. 19), to which can also be added a greater emphasis on general practice and community involvement. The role of basic sciences in PBL is still controversial, one set of critics saying that, ‘Some medical schools have now largely abandoned formal teaching of basic medical sciences’ [25], to which a reply was that, ‘PBL is *not* about sacrificing the basic sciences’ [26]. Even proponents of PBL do though recognise some potential disadvantages,

‘PBL sessions may not be structured for optimal decision making as they ask learners to construct meaning independently from data without providing guidance on optimal direction, credible references, nor guides to decision making. As such the PBL learning process is inherently exploratory and therefore inefficient. These inefficiencies highlight the downstream consequences of PBL … ’ [21](p. 138).

The literature on PBL is voluminous despite a range of reviews and meta-analyses [27–31]. However, these are not definitive on PBL’s strengths and weaknesses. As Neville said in 2009,

‘Problem-based learning (PBL) has swept the world of medical education since its introduction 40 years ago … [albeit] … *leaving a trail of unanswered or partially answered questions about its benefits*’ [32] (p. 1, our emphasis).

Recurrent suggestions are that PBL students ‘find the [ir] learning environment more stimulating and humane’ [33] [p. 564] and that after graduation, there are effects on ‘physician competencies … in the social and cognitive dimensions … [but not] in technical and teaching dimensions’ [31] [p. 40]. Much of the problem arises because many studies have considered students in only one or a few schools. Studies of the consequences of PBL have also taken little account of the possible differences between the characteristics of schools which have chosen to introduce PBL, or the students who have themselves chosen to study in PBL schools, either in terms of academic qualifications [34] or in personality or other measures [35].

### The present study

The present study uses medical school timetables to define an hour-by-hour analysis of the teaching that takes place in medical schools, allowing a detailed description of differences in UK medical school teaching, particularly considering the role of problem-based learning (PBL). The study can therefore be seen as an exercise in ‘mining the data of the multifaceted curriculum’ [36], to produce standardised ‘curriculum maps’ [37] for a majority of UK medical schools which are directly comparable between schools. Armed with measures derived from these curriculum maps, we can produce an empirical taxonomy of differences between medical schools in their teaching. The *MedDifs* companion paper [1] then goes on to analyse how differences in content and format of teaching relate to differences in medical school outcomes, including performance in postgraduate examinations, and whether doctors choose to enter general practice or psychiatry.

All courses inevitably have a timetable, so that students know what they should be doing, where and when, and together those timetables summarise student contact hours and the content of those hours, as well as the teaching formats used. The present study used the UK *Freedom of Information Act 2000* (FoI) to obtain sets of timetables from medical schools.^1^ However, timetables themselves are not always readily interpretable to outsiders, requiring local information from those within the medical schools to unpack them properly. The lead researchers therefore recruited students from different years in the various medical schools to classify and code each of the individual timetabled events within medical schools, using the timetables as a basis. The research would not have been possible without this extensive involvement of the local collaborators who were integral to the success of the study, making it appropriate that they are named here as co-authors on this paper, speaking for and validating specific data from their own medical school. We also note that such widespread authorship is now commonplace in the biomedical sciences [40]. A similar exercise in ‘citizen science’ has previously been carried out elsewhere within medicine in the *STARSurg* studies [41, 42].

The present study has the advantage of being able to compare the details of teaching within the single national system of the UK, of which ten schools out of the 25 studied here can broadly be labelled as PBL in approach. The companion study, *MedDifs*, also compares PBL and non-PBL schools in relation to measures of entry qualifications, processes within courses, student perceptions, and postgraduate outcome measures [1]. As such, it might provide the requested ‘rigorous comparison of the doctors produced by new [i.e. PBL] and traditional curriculums [ … ] which … follows doctors as they progress through their career [s]’ [25].

## Method

The core of the present study is the Analysis of Teaching of Medical Schools (*AToMS*) survey with its detailed analysis of timetabled teaching events.

### Timetabled teaching events in AToMS

We used a collaborative approach to data analysis, utilising the resources of the Medical Student Investigators Collaborative (MSICo) for the labour-intensive task of coding each timetabled activity in a standard format. Although clinical timetables may seem simple, in practice, they need interpretation, and therefore, local analysis teams were recruited from each school to interpret the complex nature of the timetables obtained and code them in a standard format, including the length, the teaching format, and the teaching content of each session. Standardisation was assisted by using a term book, with individual questions adjudicated consistently by Oliver Devine. Teaching formats were classified into 20 different categories, and most teaching sessions could be allocated to one of these categories. Teaching content was firstly coded using whatever phrase was used in the timetable itself, with over 70 different terms being found, the terms subsequently being composited into 18 groupings to take account of likely synonyms. Start and end times were recorded for each teaching event, along with duration (which allowed for error checking).

### Self-directed learning and self-regulated learning

For this study, we consider time for *self-directed learning* to be that specified (‘directed’) as such in medical school timetables and which has a clear duration; it will later be seen that it is present in all but one medical school. Self-regulated learning, in contrast, is ‘regulated’ by students themselves and can only be quantified by self-report as in two studies [39, 43]. We acknowledge that neither self-directed nor self-regulated are entirely satisfactory terms.

### Names of medical schools

Research papers often use inconsistent names and abbreviations for medical schools. Here, we have names based on those used by the UK Medical Schools Council (MSC) [44]. More details of all schools can be found in the World Directory of Medical Schools [45].

### Medical schools

In 2014–2015, there were 33 medical schools in the UK. Our analysis of teaching considers only schools which have 5-year (standard entry) courses for undergraduates, and therefore, Warwick and Swansea medical schools which are graduate entry only are not included. Where schools have both 5-year and graduate entry or other courses, we only consider the 5-year course. Standard entry courses were provided by 31 schools, of which data were available for 25 schools (Aberdeen, Barts, Birmingham, Brighton and Sussex, Cambridge, Cardiff, Dundee, Edinburgh, Glasgow, Hull York, Imperial, Keele, King’s, Leeds, Leicester, Liverpool, Manchester, Newcastle, Norwich, Nottingham, Oxford, Queen’s, Sheffield, St George’s, and UCL). Six schools were omitted from the study: Exeter and Plymouth as they were reorganising after Peninsula Medical School was split, St Andrews as it does not have a clinical course, Lancaster as it has only recently produced graduates, and Bristol and Southampton for logistical reasons.

### Problem-based learning schools

A useful distinction is between schools that are or are not regarded as PBL. There is no hard classification, and for convenience, we use the classification provided on the BMA website for the eleven UK schools described as either PBL or CBL (case-based learning)^2^, i.e. Barts, Cardiff, Exeter, Glasgow, Hull York, Keele, Liverpool, Manchester, Norwich, Plymouth, and Sheffield [47], with the addition of St George’s whose students and website described the school as PBL. Ten of these PBL schools are in the 25 schools studied here.

### Medical school year numbering

Medical school year numbering is not always consistent, some medical schools having compulsory intercalated/integrated BSc or other degrees. For present purposes, intercalated years were omitted, and other years labelled as years 1 to 5. Many schools refer to years 1 and 2 as basic medical sciences (BMS) and years 3, 4, and 5 as clinical (Clin). It is recognised that this is not always an accurate description of course content for some medical schools which have more integrated courses. We therefore simply refer to years 1, 2, 3, 4, and 5.

### Other datasets

We have used three external datasets to validate aspects of the current data or to contribute to the analyses. In particular, we are grateful for having been given access to the following: the HEPI datasets which annually ask a representative set of students at UK universities to complete a questionnaire about their teaching, data from a study which asked UK medical students about self-regulated teaching time [39], and a study of teaching of general practice which collected data from heads of Departments of General Practice in UK medical schools [48].

### The level of analysis

It must be emphasised that throughout this study, all measures are at the level of medical schools and are not based on raw data at the student level. It is likely that students vary in the extent to which they attend provided teaching, and we have no direct data on that.

### Statistical analysis

The majority of conventional descriptive and inferential statistics were calculated using IBM SPSS v24. Factor analysis was used to explore the inter-relations of the various measures and to reduce them to a smaller set of more informative measures. R v3.4.2 [49] was used to carry out the factor analysis, in particular using Velicer’s parallel analysis in the *fa.parallel()* function in the *psych* package for deciding on the number of factors, and calculation of normal (van der Waerden) scores with *score()* in the *jmOutlier* package to convert non-normal distributions to normal scores. Some plotting used *ggplot2()* in R.

### Ethical permission

None of the data in this study are personal data, the data only relating to administrative data on medical school timetables, and therefore, ethical permission was not required.

## Results

A total of 47,258 timetabled events were recorded at 25 different UK medical schools for the 2014–2015 academic year, with a mean of 1890 events per school (SD = 342, range = 1302 to 2616). Overall, the numbers of events classified for each year were 8996 (year 1, 19.0%), 8402 (year 2, 17.8%), 11,253 (year 3, 23.8%), 10,176 (year 4, 21.5%), and 8381 (year 5, 17.7%). Elective and SSC (student-selected component) hours were not classified by year.

### Teaching format and duration

Teaching events differ in their format and are broadly classified as formal teaching (*n* = 43,317), timetabled self-directed learning (*n* = 3341), student-selected components (SSCs; *n* = 25), electives (*n* = 25), and unspecified (*n* = 550). SSCs and electives were recorded as a single teaching event per school, so that the mean length is long (SSCs—408 h, SD = 202 h, range = 70 to 735 h; electives—259 h, SD = 42 h, range = 175 to 350 h). Excluding SSCs and electives, timetabled teaching events had a mean duration of 2 h 6 min, a median duration of 1 h 30 min, and a modal duration of 1 h 0 min, with a standard deviation of 1 h 23 min and 95% range of 30 min to 4 h 30 min, skewed to the right (skewness = 1.51) with a minimum of 5 min and a maximum of 25 h 15 min which was a clinic session in the Emergency Department.

### Start and end times

**Fig. 1:**
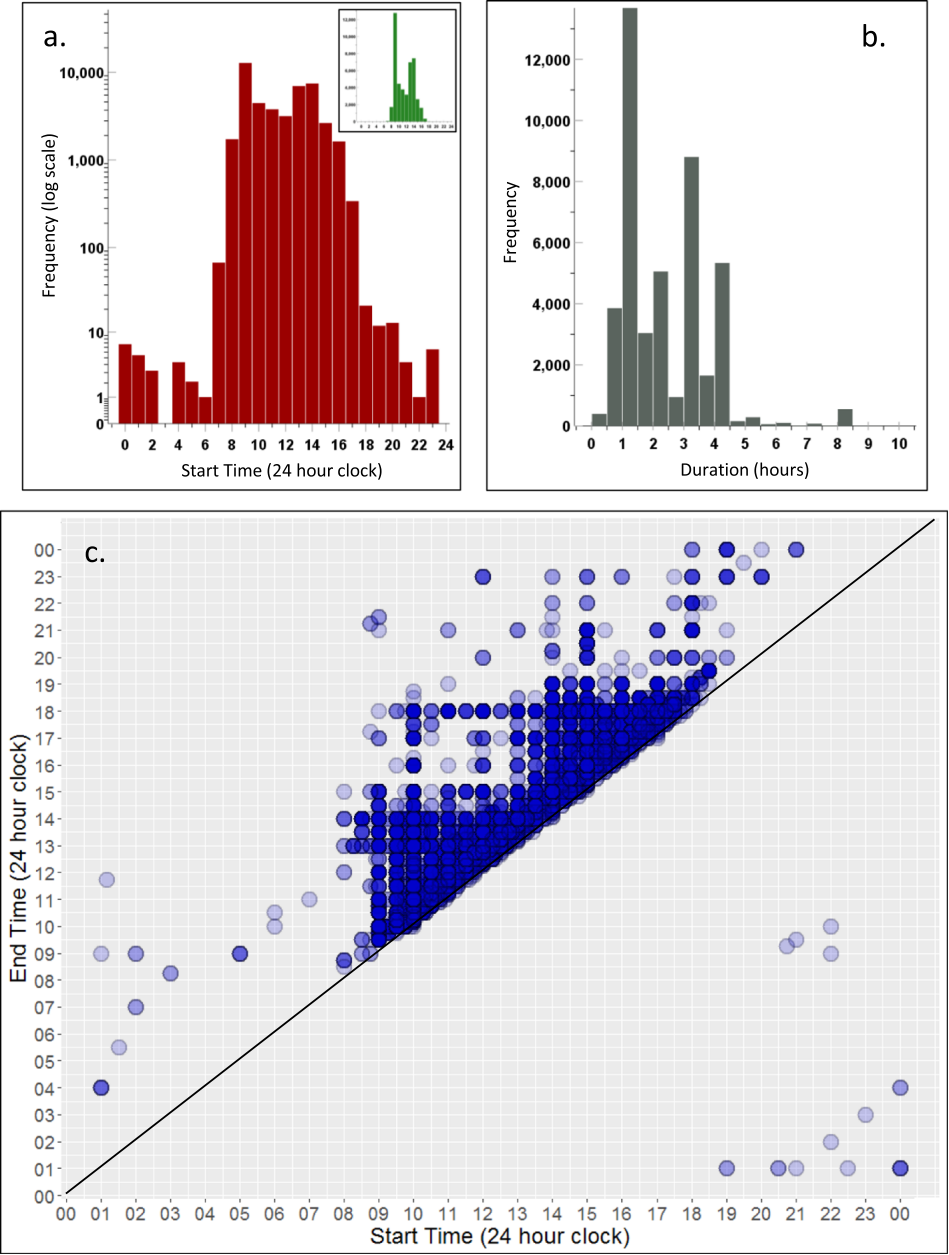
Start and end times of teaching events: **a** start time on logarithmic scale (red) (inset: start time on linear scale (green)), **b** duration in hours (grey), and **c** start and end time (blue). In **c**, note that some events start on 1 day and finish on the next

Timetabled events typically have a modal length of 1 h and start during normal working hours (mean = 11:33, median = 11:10, mode = 09:00, with a 95% range from 08:30 to 16:00; there are visible modes at 09:00 and 13:00–14:00). However, as Fig. 1a–c shows, a small proportion of events occur outside of normal working hours. The scattergram of end time in relation to start time shows that some teaching occurs during the evening, night, and early morning, and can be of long duration, as would be expected with clinical teaching.

### Durations of timetabled teaching events

**Fig. 2:**
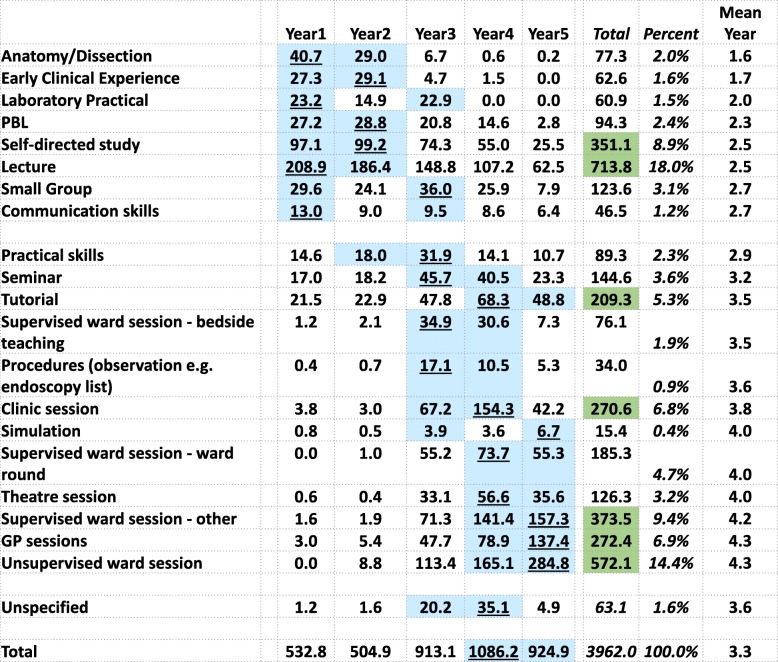
Average hours of the different teaching formats for a student at a typical medical school by medical school year. For each teaching format, the 2-year groups with the highest amount of teaching are in blue, with the highest value underlined. Green shading denotes totals of over 200 h. The two groups of teaching formats designate those that are mostly basic medical sciences and mostly clinical, respectively

**Fig. 3:**
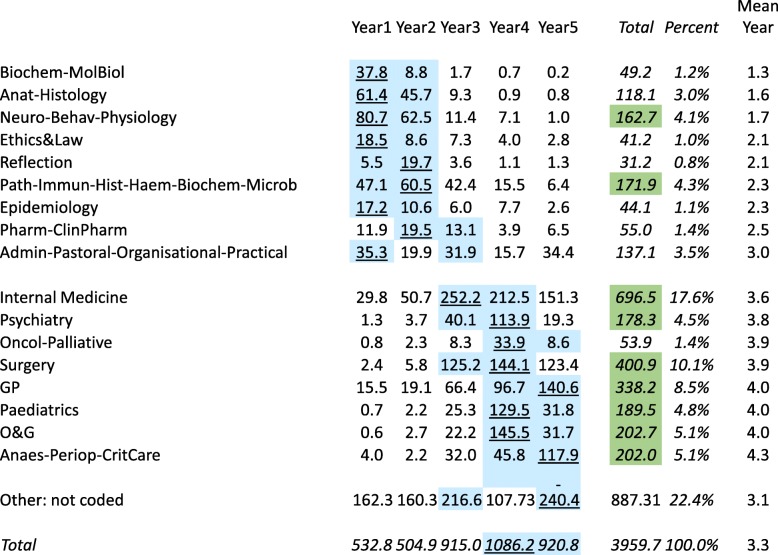
Average hours of teaching for the different teaching contents for a student at a typical medical school by medical school year. For each teaching content, the 2-year groups with the highest amount of teaching are in blue, with the highest value underlined. Green shading denotes totals of over 150 h. The two groups of teaching contents designate those that are mostly basic medical sciences and mostly clinical, respectively. Note that SSCs and electives are not included as they are not allocated to particular years

Although the basic unit of analysis is the timetabled teaching event, some events are much longer than others. A simple count of number of events does not take event length into account, therefore making results difficult to interpret. To express the data in a clearer way, we have therefore weighted teaching event data by the length of the event. In Figs. 2 and 3, the times have also been divided by 25, the number of medical schools in the study, and the tables can therefore be read directly as *the total number of timetabled teaching hours experienced by a typical medical student at a typical medical school* for each teaching format or content, either within a year or within the entire course. Teaching times in year 1 and year 2 average 518.9 h, which for a notional teaching year of 30 weeks is 17.3 h/week, whereas the mean time for years 3, 4, and 5 is 974.7 h, which for a typical year of 48 weeks is 20.3 h/week. The overall total teaching time is 3962 h, which excludes SSCs and electives, which had estimated mean total times of 408 and 259 h, so that the total of all teaching time for an average medical student is 3962 + 408 + 259 = 4629 h.

### Teaching formats

Timetabled events were classified into twenty different teaching formats. Figure 2 shows the number of hours of each format of teaching experienced by a typical medical student for each of the five course years, sorted by the mean year of the course in which the format is used. There is a cluster of teaching formats used mainly in the first 2 years, typical of BMS teaching, and then a second cluster of teaching formats in years 3, 4, and 5, mainly consisting of clinical teaching methods. Lectures predominate across the course as a whole with a mean of 714 h, 18% of all teaching. Timetabled self-directed study has 351 h and occurs in all years, but particularly years 1 and 2. Within years 3, 4, and 5, unsupervised ward sessions account for 572 h, followed by supervised ward sessions—other (373 h), GP sessions (272 h), and clinic sessions (271 h).

### Teaching content

Classifying teaching content was difficult, not least because some medical schools teach more integrated courses than others, and also the same topic can often be named in different and overlapping ways (e.g. biochemistry or molecular biology). Overall, there were over 70 specific terms used, with some restricted to one or two medical schools. After several exploratory attempts, the different terms for teaching content were agglomerated into 18 conceptually distinct categories, which are shown in Fig. 3. The figure is sorted by the mean year in which teaching typically occurs. A broad separation occurs between teaching content typically taught within years 1 and 2 and teaching content taught more within years 3, 4, and 5. Within years 1 and 2, pathological sciences (171 h), neurosciences/behavioural sciences/physiology (163 h), anatomy/histology (118 h), and pharmacology/clinical pharmacology (55 h) are the classic ‘pre-clinical’ or basic medical sciences. Other topics typically taught in years 1 and 2 include reflection (31 h), ethics and law (41 h), and epidemiology (44 h). Years 3, 4, and 5 are dominated broadly by clinical topics, by internal medicine (696 h), followed by surgery (401 h) and general practice (342 h). Psychiatry (178 h), paediatrics (190 h), obstetrics and gynaecology (203 h), and oncology/palliative care (54 h) are characterised by occurring mainly in year 4, while anaesthetics/perioperative care/critical/emergency care (202 h) is the only topic occurring mainly in year 5. Some ‘clinical’ topics do occur in years 1 and 2, notably internal medicine (30 h in year 1) and general practice (16 h in year 1). Administrative/pastoral/organisation/practical topics (137 h) occur across the entire course. Finally, the inevitable arbitrariness and difficulty of any classification is shown by the 887 h, 22% of all teaching, for which coders were unable to make specific attributions to any one dominant content area. As will be shown later, these hours are much more likely to occur in PBL courses, and in part reflect the nature and flexibility of PBL teaching itself.

### Differences between medical schools

**Fig. 4:**
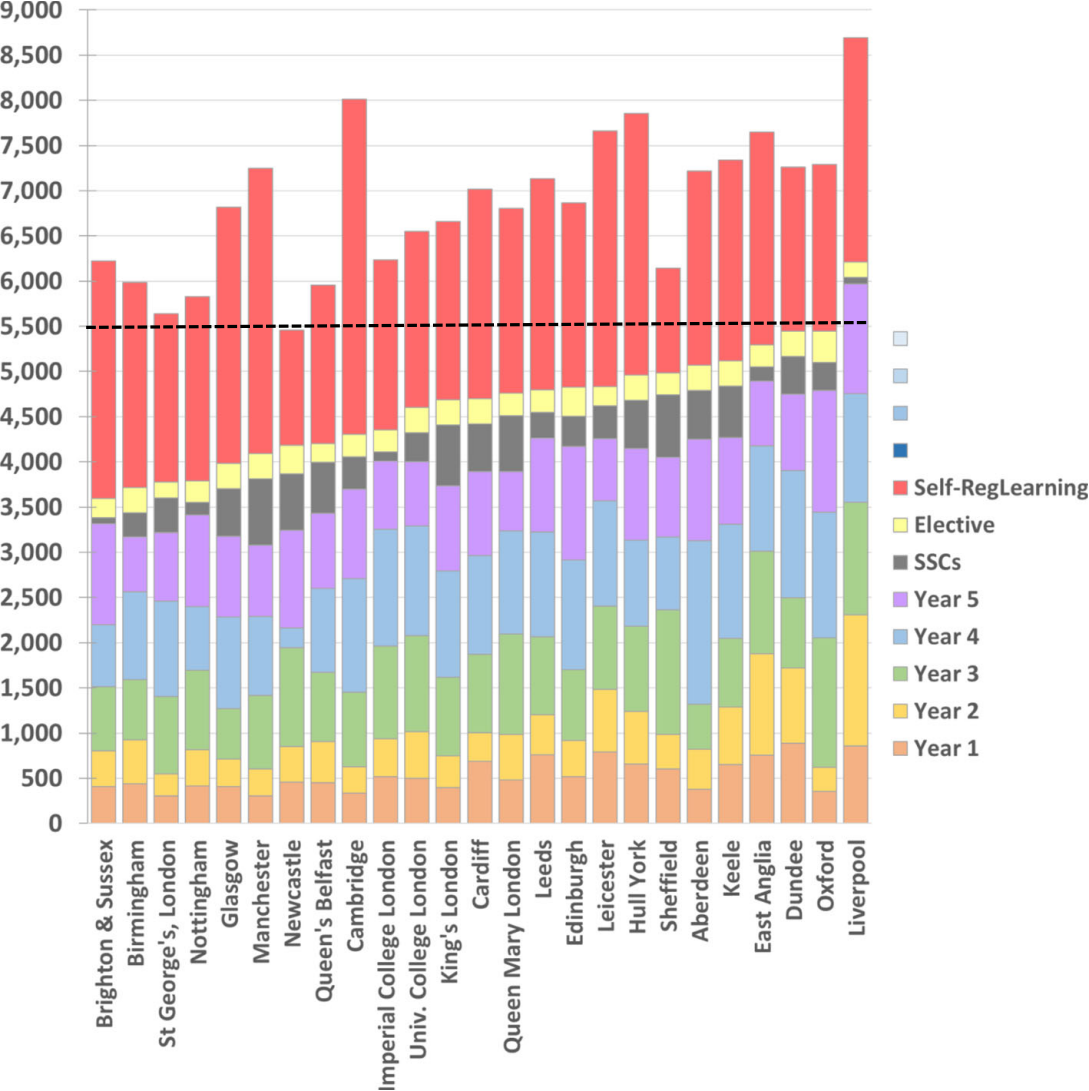
Total hours of teaching at the 25 UK medical schools. Times are stacked for years 1, 2, 3, 4, and 5, followed by SSCs and electives, all based on the *AToMS* survey. Schools are sorted by total teaching time in the *AToMS* study. These are followed by estimates of self-regulated learning; see text for details

Figures 2 and 3 have given an overall view of the pattern of teaching in UK medical schools for a typical student, but a primary interest of the survey is in differences between medical schools. Figure 4 summarises the total hours of teaching in each school, broken down by year, with a range of 3593 to 6213 h for formal teaching, excluding SSCs and electives which are shown separately. For details of estimates of self-regulated learning, see the end of the “Results” section.

**Fig. 5:**
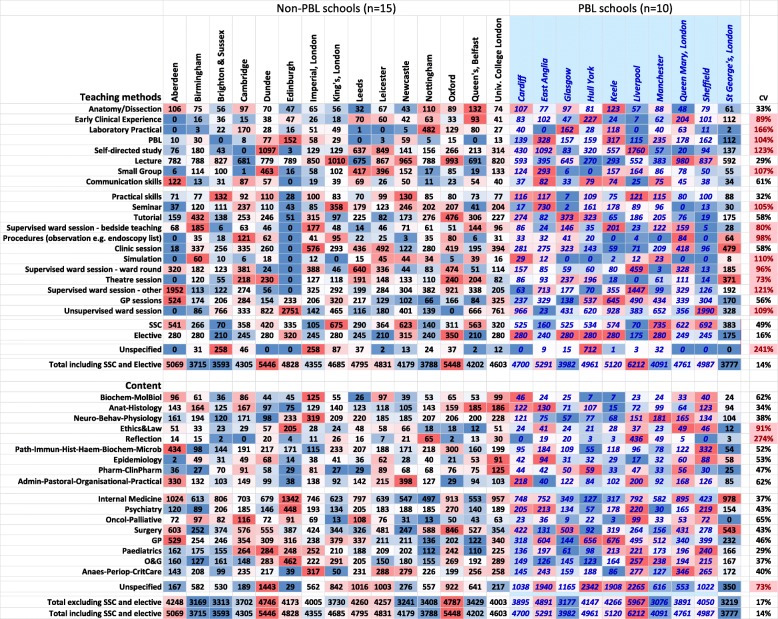
Teaching at individual medical schools. Number of hours of teaching in terms of teaching format (upper) and teaching content (lower). Format and content are ordered in the same way as in Figs. 1 and 2. Medical schools are structured in terms of non-PBL and PBL schools, with schools sorted alphabetically within groups. Within entire rows, colours indicate the highest number of teaching hours (red) and the lowest number of teaching hours (blue). The final column marked CV shows the coefficient of variation; values > 80% are shown in red

**Fig. 6:**
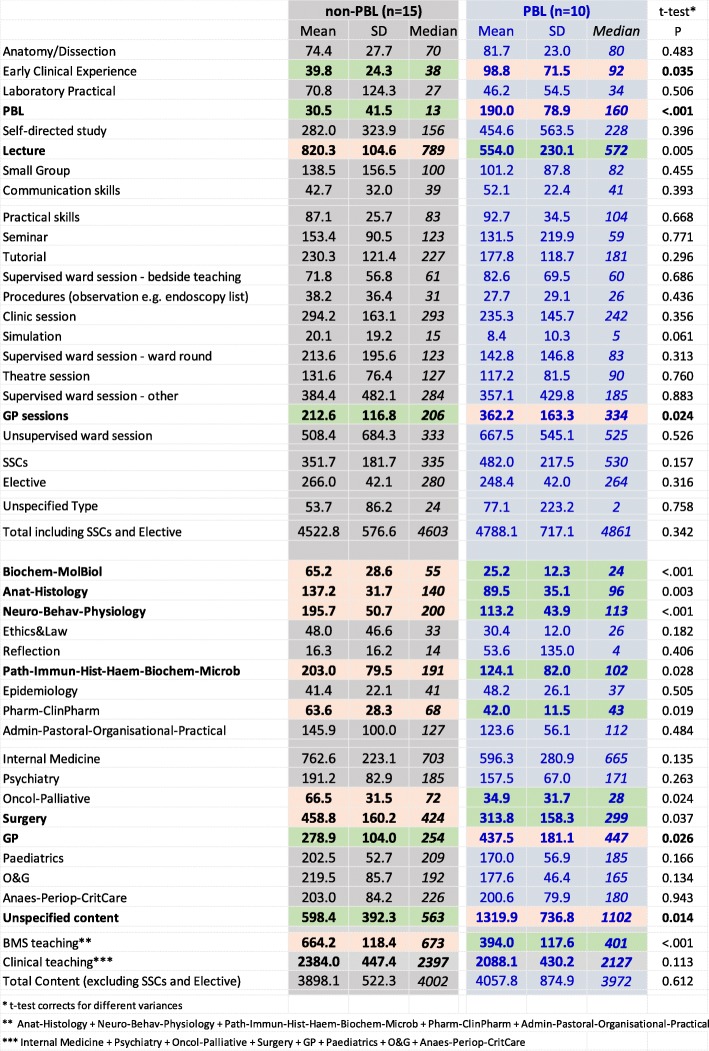
Teaching formats and contents at PBL and non-PBL schools. Average (SD; median) hours of teaching for the different teaching format and content areas for an average student at the ten PBL schools and the fifteen non-PBL schools. Differences significant on the *t* test (*p* < .05) are shown in colour, red indicating the group with the greater amount of teaching and green the lesser amount of teaching. *t* tests take account of differing variances, and significant results are shown in bold

Differences in the details of teaching at each school are summarised in Fig. 5, with PBL and non-PBL courses separated. Teaching format and teaching content are shown together, as often these might be expected to be interlinked (e.g. anatomy/dissection in teaching format with anatomy-histology in teaching content). The data for Fig. 6 are available as a spreadsheet in Supplementary File 1.

**Fig. 7:**
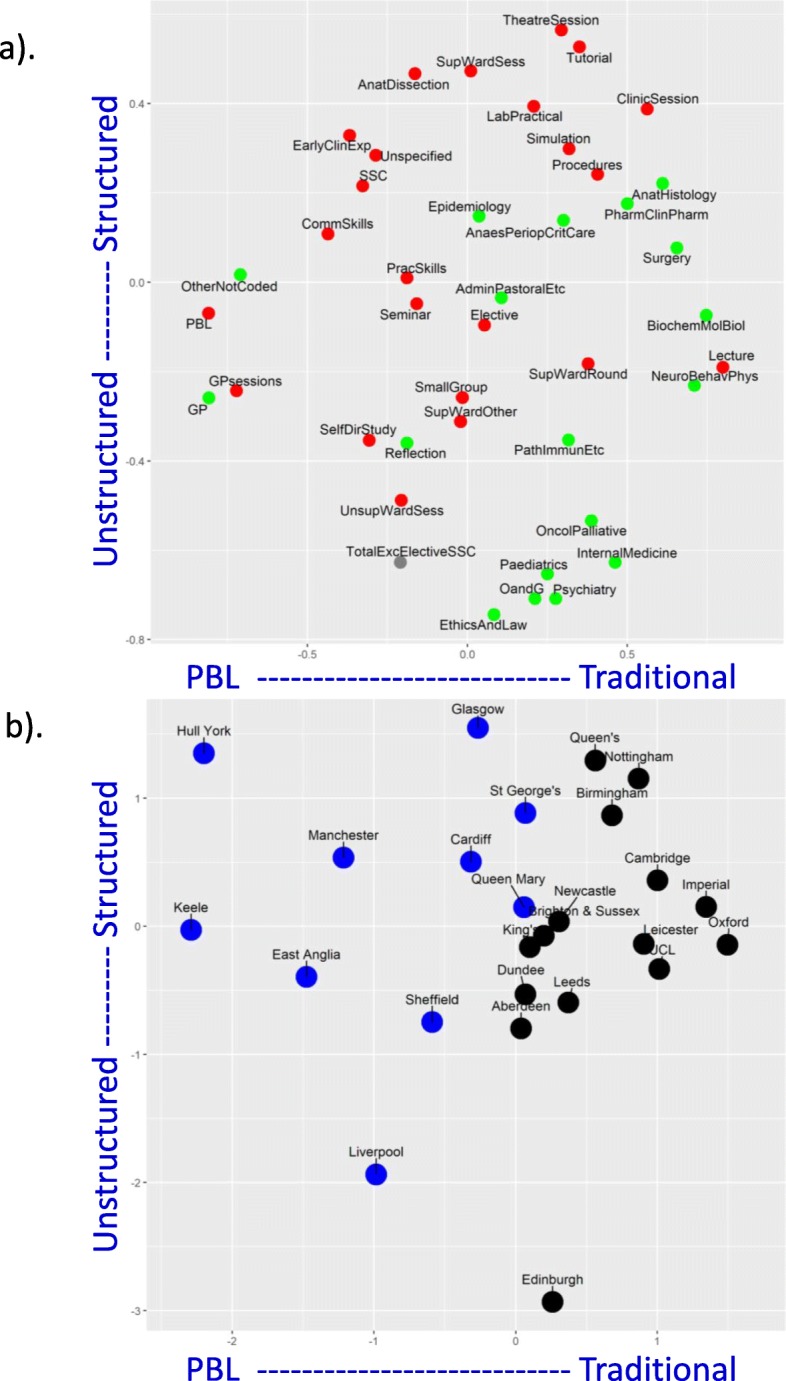
Curriculum map of formats and contents. **a** Top: loadings of teaching measures on the first two factors with format measures in red and content measures in green. **b** Bottom: scores of medical schools on the first two factors: blue—PBL schools, black—non-PBL schools

**Fig. 8:**
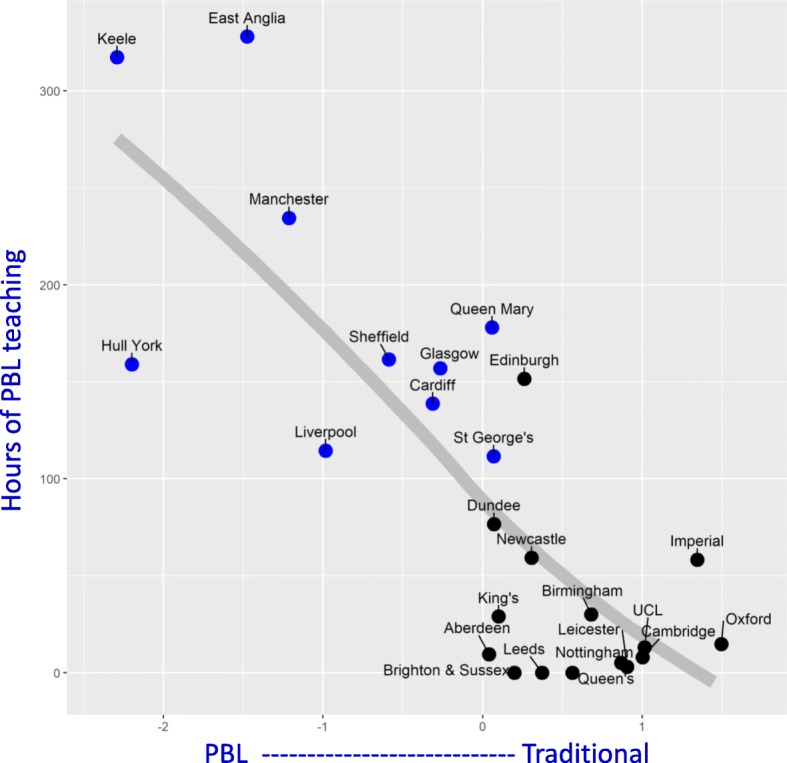
Hours of PBL teaching for individual medical schools. Scores of PBL (blue) and non-PBL schools (black) on the first factor (PBL vs traditional) in relation to timetabled hours of PBL teaching (vertical). The fitted line is a Loess curve

Figure 5 is complicated, but emphasises the variation in how different medical schools organise and describe their teaching, and that itself belies any simplistic, unitary description of ‘UK Medical Education’. In navigating through Fig. 5, some comments may be helpful:

1. *PBL schools.* Medical schools can be broadly divided into those which do or do not principally use PBL, and ten schools were classified as PBL schools (see the “Method” section). The PBL schools are shown to the right in Fig. 5 with a blue, italic font. Figure 6 compares the numbers of hours for each of the teaching formats and contents of the PBL and non-PBL schools. Some measures have wide variation, and differences in variance are taken into account in the *t* tests. Fifteen of 45 differences (33%) are significant with *p* < 0.05, and four are significant with a Bonferroni-corrected significance of .05/45 = 0.0011. PBL schools have *more* hours of PBL teaching, early clinical experience, sessions in general practice, GP teaching, and unspecified content. PBL schools also had *fewer* hours in lectures, biochem-molecular biology, anat-histology, neuro-behav-physiology, pathology etc., oncology-palliative care, and surgery. The five main BMS subjects (biochemistry etc., anatomy etc., neuroscience etc., pathology etc., and pharmacology etc.) accounted for fewer hours overall in PBL schools, but there were no differences in total teaching in the eight clinical topics.
2. *Measures with greater variability.* Occasional rows in Fig. 5 have a large variability, a good example being laboratory practicals for Nottingham, which with a value of 482 is much larger than most other medical schools. Variability was assessed systematically as the percentage coefficient of variation (CV) across medical schools, calculated as 100 × (SD scores)/(mean of scores). The mean (median) CV across all measures is 73% (58%). CVs are shown in Fig. 5, with red shading indicating CVs greater than 80%. Overall, there is much more variation across medical schools in formats of teaching rather than content of teaching, although a major exception is ‘reflection’, which receives 436 h at Liverpool, but the second highest value anywhere else is 65 h, at Nottingham, the CV being 274%. Amongst formats of teaching, laboratory practicals showed the most variability (166%), followed by self-directed study (123%) and supervised ward session—other (121%). Noteworthy is that total teaching times showed least variability (17% and 14%) suggesting that variation between schools was because schools mostly chose to allocate time differently, not because they had different overall teaching times.
3. *Factor structure of medical school teaching.* The complexities of Fig. 5 have been reduced by using a principal component analysis of the 42 measures (the totals having been excluded since they are redundant). The correlation matrix is necessarily singular, there being 42 measures but only 25 schools, but a principal component analysis can still be carried out. A concern is that a number of the measures in Fig. 5 are skewed, and therefore, all measures were converted to normal (van der Waerden) scores. Velicer’s parallel analysis suggested there were three significant factors, but reification of all the factors was not straightforward, and therefore, for simplicity, only the first two factors were extracted, without rotation, which accounted for 31% of the total variance. Factor scores for the individual schools were extracted using the regression method. Factor 1 is labelled *Traditional* vs *PBL teaching*, and factor 2 is labelled *Structured* vs *Unstructured teaching*.
  Figure 7a shows the loadings of the teaching format and teaching content measures on the first two factors. The first factor, *Traditional* vs *PBL teaching*, has loadings to the left-hand side on PBL teaching time, as well as GP sessions, and loadings to the right-hand side on lectures, biochemistry etc., neuroscience etc., anatomy-histology, surgery, and internal medicine. This factor is clearly distinguishing PBL courses from traditional courses. That is strongly supported by Fig. 7b which shows the factor scores for each medical school on the two dimensions, with PBL and non-PBL courses plotted separately. The ten PBL schools in blue are distinct as a group from the non-PBL courses (in black), although there is an area of overlap in the middle. The major predictor of *Traditional* vs *PBL teaching* is hours of PBL teaching, and Fig. 8 shows the close relationship. Nevertheless, in both Figs. 7a and 8, it is clear that within both PBL schools and non-PBL schools, there is variation on PBL hours and *Traditional* vs *PBL teaching* scores, suggesting a continuum of the extent to which schools use a PBL approach. In Fig. 7b, it is apparent that Edinburgh is clustering with PBL schools, albeit at the lower of PBL hours, and we note that its current website does refer to its PBL teaching [50], showing the inevitable arbitrariness of any hard classification.
  The second factor in Fig. 7a, b, *Structured* vs *Unstructured teaching*, is clearly separate from *Traditional* vs *PBL teaching*, and it is noteworthy in Fig. 7b that *Structured* vs *Unstructured teaching* is independent of being a PBL course, there being clear variation within both PBL and non-PBL courses. *Structured* vs *Unstructured teaching* is mostly but not entirely associated with teaching formats, the formats at the top of *Structured* vs *Unstructured teaching* in Fig. 7a including tutorials, anatomy dissection, theatre sessions, laboratory practicals, simulation, bedside teaching, observation of procedures, and clinic sessions, whereas loadings at the bottom of the figure are mainly associated with GP sessions, unsupervised ward sessions, ethics and law, small groups, reflection, and self-directed study. This factor probably relates to the extent to which teaching is organised or self-directed (although lectures do not fit well in that classification). Content areas also vary on the *Structured* vs *Unstructured teaching* factor, with anatomy being highly structured and ethics and law highly unstructured.

### Validation of estimated teaching hours against external data

The data in Figs. 2, 3, and 5 show the estimated hours of various teaching formats in different medical schools based on teaching events derived from timetables. Despite their seeming face validity, it is important to validate the measures against other data on differences in medical school teaching. Unfortunately, such data are rare, but here, we describe validation against two other estimates of teaching time.

**Fig. 9:**
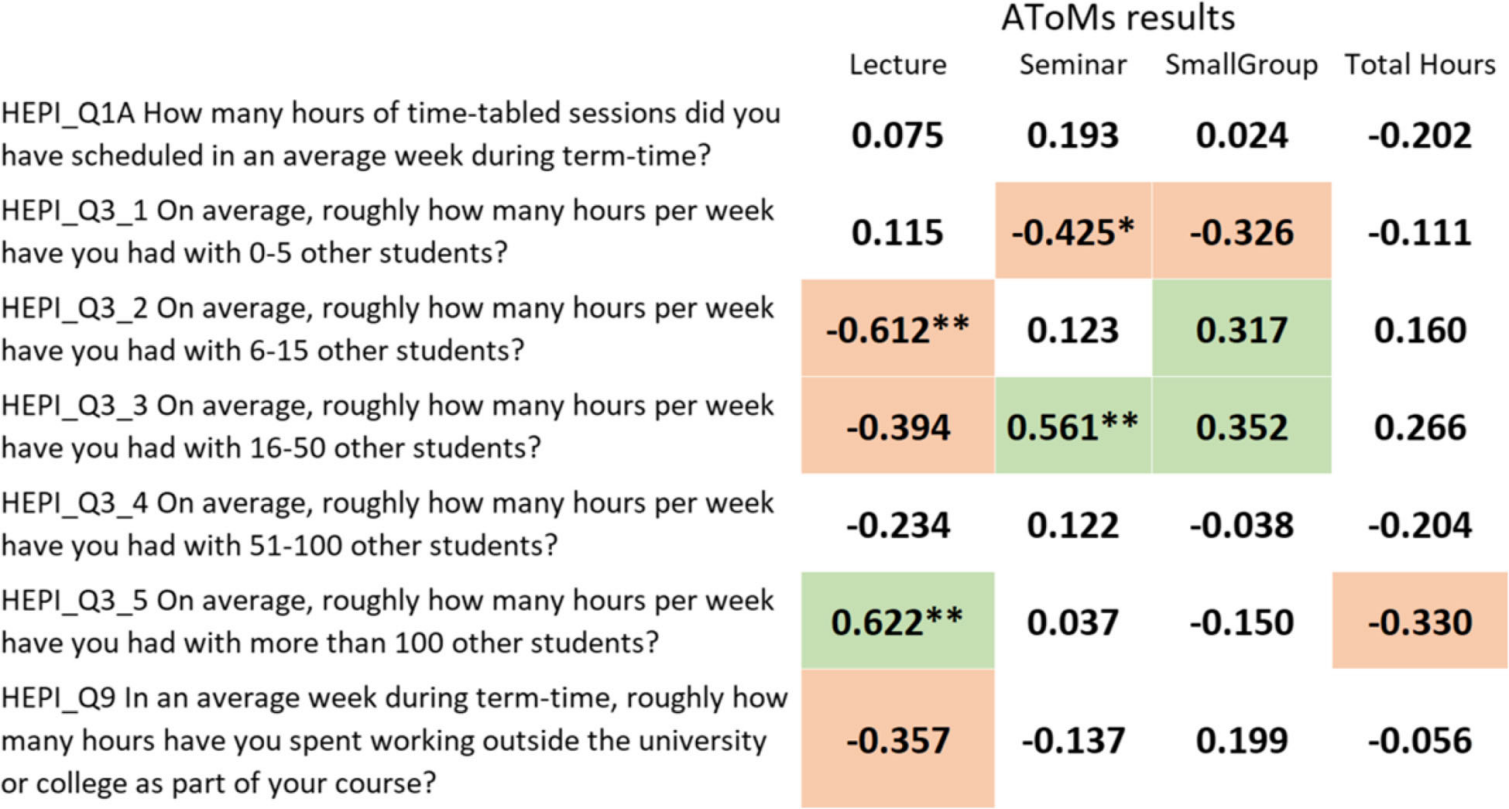
Validation of hours of teaching in the Teaching Survey with hours of teaching in the HEPI Student Academic Experience Survey. Pearson correlations based on 24 medical schools. *p < .05; **p < .001. Correlations greater than an arbitrary level of 0.3 shown in green and correlations less than an arbitrary level of − 0.3 shown in red

1. The HEPI *Student Academic Experience Surveys*. Although differences have been shown in teaching hours across different schools, that does not necessarily mean that students themselves perceive those differences. A useful comparison therefore is with the estimates of perceived contact hours in the HEPI *Student Academic Experience Surveys.* Medical students in the HEPI surveys were asked about timetabled sessions per week, both overall, and also in teaching groups of size 0–5, 6–15, 16–50, 51–100, and 100+ other students. Figure 9 shows correlations between the HEPI estimates and those for lectures, seminars, small groups, and total teaching hours for the medical schools in the current survey, with larger positive correlations in green and larger negative correlations in red. Although the total estimates in the two sets of data (HEPI_Q1A and total hours) show only a weak and negative correlation (*r* = −.202), much clearer is that student estimates of time in large groups (100+) show a strong positive correlation with timetabled lecture times (*r* = 0.622), timetabled seminars correlate positively with time in groups of 16–50 students (*r* = 0.561), and timetabled small groups correlate positively with time in groups both of size 6–15 (*r* = 0.317) and 16–50 (*r* = 0.352). The overall HEPI estimate of ‘timetabled sessions’ is perhaps too broad a measure, confounding different formats of teaching making it hard for students to answer. However, the estimates for the HEPI groups of size 6–15, 16–50, and 100+ differentiate clearly between timetabled small groups, seminars, and lectures in the AToMS data. These data therefore provide mutually supporting evidence for the validity of both the AToMS timetabled teaching event data and the perceptions of teaching load by the HEPI student respondents.
2. *Estimates of GP teaching time.* A recent study of GP teaching by Alberti et al. [48] estimated time for what it called ‘authentic GP teaching’, defined as ‘teaching in a practice with patient contact, in contrast to non-clinical sessions such as group tutorials in the medical school’. Information was provided by the current heads of GP teaching at UK medical schools for students entering in 2007 and 2008 (for which no differences were described). Schools in the Alberti et al. paper were not named, but we are grateful to the authors for providing us with raw data on total GP teaching time and authentic GP teaching time. For our own data, we calculated an equivalent to the authentic teaching score by considering only teaching described as clinically based within GP. For the 25 schools in our study, total GP teaching correlated 0.692 (*p* < 0.001, *n* = 25) with the total teaching time estimates for the same schools in the Alberti et al. study, and estimates of authentic teaching in our study correlated 0.709 (*p* < 0.001, *n* = 25) with the estimates from the Alberti et al. study. Authentic teaching represented about 77% of all GP teaching in our data and about 82% in the Alberti et al. data. The total duration and the proportion of authentic teaching are similar in our study and that of Alberti et al. The data from the two studies are therefore reassuringly similar, despite being estimated in different ways.

Together, the HEPI and the Alberti et al. data provide a good validation of the teaching times estimated using our own methodology and provide reassurance of the other estimates of teaching time.

### Estimating hours of self-regulated learning

The *AToMS* study only includes time for *self-directed learning* where it is explicitly directed in medical school timetables (which itself may be somewhat oxymoronic). Medical students are also, however, expected to study in their own time, which we distinguish from self-directed learning by calling it *self-regulated learning*, as it is regulated by students themselves. We know of two UK studies which have estimated *self-regulated learning*, the study of Lumley et al. [39] which had data from 20 UK medical schools and the HEPI study which included all UK medical schools. For the 20 medical schools with data in both studies, the correlation was 0.515 (*p* = 0.020; alpha reliability = 0.67). It should be noted that ‘time-logging’ data suggest that in general, student estimates of time spent on academic activities correlate well with actual time spent [51], suggesting that the data from the two studies are likely to be valid estimates of actual time. Data from the two studies were merged by converting mean time at each of the 29 medical schools to a *z*-score, averaging the *z*-scores if there were two estimates, converting the final values to *z*-scores, and then back-estimating actual hours based on the mean and SD in the Lumley et al. study, which had explicitly surveyed medical students. For the 25 schools in the current study, the estimated means of self-regulated learning by medical school varied from 5.7 to 18.2 h per week (mean = 11.2, SD = 3.02; *N* = 25 medical schools). On the basis of two pre-clinical years of 30 weeks, and three clinical years of 48 weeks, these times are multiplied by 204 and included in the stacked bar chart of Fig. 4 as red bars. It is worth noting that the average self-regulated learning across the course (11.2 × 204 = 2284 h) is equivalent to about 49% of the average formal timetabled teaching (4629 h, including SSCs and electives), as can be seen in Fig. 4, confirming that much student study and learning take place outside of formal teaching.

## Discussion

The *AToMS* study provides what is perhaps the first comprehensive timetable-based analysis of variation in teaching formats and contents in the majority of UK medical schools, with possible predecessors in the 1975 and 1988 surveys of UK medical schools by the General Medical Council [52, 53], which though are discursive and more limited quantitatively. In contrast, our data are quantitatively rich and raise a number of issues which we consider in turn.

### The role of the GMC

In 1957, the GMC, which had been created 99 years earlier,

‘abandoned the principle of recommending a prescribed minimal curriculum to the medical schools … Instead it issued ‘Recommendations’ which were most permissive, reminded the schools that they were responsible for designing their own curricula, and exhorted them to experiment’ [54].

In the years that followed, how and what teaching was actually taking place in each medical school became far less clear, despite many undoubted changes in medical school curricula [55]. Liberalisation mainly followed on from *Tomorrow’s Doctors* in 1993, but it is far from clear what the effects were. That problem mattered relatively little until the past decade when pressure from the NHS and HEE forced questions to be asked about the effects of different formats of medical training, with answers in short supply. The research solution required data from medical schools, but historically, medical schools have been reluctant to publish data which might allow differences between them to be inferred, as notionally all are equivalent via GMC accreditation. However, indirect evidence has slowly emerged over the years suggesting that any idea of equivalence was incorrect [56–58]. The time has come, as the GMC itself realises [59], for proper comparative data from medical schools to be made available.

The GMC, in the context of a report on the extent to which medical students are prepared for foundation practice, has overviewed medical school differences quite generally [60]. It began by saying that:

‘Variation between medical schools in the interests, abilities and career progression of their graduates is inevitable and not in itself a cause for concern … ’

Inevitably a statement such as that is followed by caveats, and the overall tenor of the report is that medical school differences do matter, or at least might matter. We consider the relationship between medical school teaching differences and a range of other measures such as the qualifications of entrants, the resources available, the perceptions of teaching, and the outcomes in foundation training and postgraduate examinations in the *MedDifs* study [1]. The purpose of the present study is to provide a conceptual map of medical school teaching and the differences that occur, with the impact of those differences considered later [1].

### Obtaining information from medical schools

The majority of medical schools collaborated with our study, and we thank them very much for their assistance. We hope that the details described in the comparative data presented here will justify their time and effort in contributing to an unusual and important study. That a minority of medical schools refused to provide information on a topic as basic as the teaching that they provide was disappointing.

### Limitations of the data available in the present study

Medical school curricula are complex, and different people may well describe the same events in different ways. We have attempted to describe the teaching formats and teaching content of timetables, and no doubt that could have been done differently. Despite standardisation of our coding definitions across our team of coders, precise distinctions between tutorials, seminars, and group work are not always possible, and different schools may use the same terms in different ways. Teaching on subjects such as ‘molecular biology’ or ‘paediatrics’ may be ostensibly of the same length but contain very different material. Indeed, different students at a single medical school will inevitably have different content in their teaching, particularly in clinical subjects, and of course, even if students attend the same teaching, it does not mean that they equally are interested by, attend to, or retain that content. There is no doubt our study could have been done differently and in much greater depth. We are nonetheless gratified that our two validation tests—with the HEPI data and with data on GP teaching in medical schools—find that our results are corroborated by other studies. We therefore believe that this study is a starting point for future studies which can look in further detail both at individual teaching contents, and the broader picture of medical school teaching, perhaps carried out on an official basis.

### Total teaching time at UK medical schools and the European Directive

Although the primary interest of our study was not in total teaching time, our study nevertheless provides useful information. The Medical Act 1983 does not specify a specific duration for a medical course, but European Directive 93/16/EEC specified that 5500 h of ‘theoretical or practical instruction’ should take place ‘under the supervision of a university’ before the completion of undergraduate medical training. The Directive also specified a minimum of 6 years for the course, which resulted in what has been called a ‘legal fiction’ that the first foundation (pre-registration) year was a part of the course. The requirement of 6 years was subsequently removed by Directive 2013/55/EU.

Figure 4 shows the total volume of timetabled teaching events at each school, with a range of 3543 to 6205 h from the least to the greatest, giving a coefficient of variation of 14.4% (mean = 4569, SD = 657). It should be noted that intercalated/integrated BSc/BMedSci/BA degrees are not included in these totals, although such degrees are compulsory at Oxford, Cambridge, Imperial College, UCL, and Nottingham.

We are also aware that even when self-directed study is not timetabled in some medical schools, there is nevertheless an expectation of additional work which would come under the heading of self-regulated learning, and should be added to the total hours that can be regarded as education in a broader sense.

Self-regulated learning, in one study of UK medical students, averaged 10.6 h per week during term time [39], a figure similar to the 9.8 h reported by clinical students in Porto in Portugal [43]. A slightly higher value was reported in the HEPI data, with a mean self-reported independent study (private study) of 16.3 h per week (question Q7; SD = 10.7, *N* = 2657 medical students). Estimated hours of total self-regulated learning, as described in the “Results” section, are included within Fig. 4. We realise that there is a possibility of double counting the self-directed study that is explicitly written into timetables and the self-regulated/independent/private learning which occurs but is not directly prescribed by medical schools. It is also possible that some schools have additional hours, not captured in our survey because they are not written down in timetables. Nevertheless, the estimates are useful and should encourage further research on the topic.

The European Directive time of 5500 h does set a useful yardstick against which to compare medical school teaching, and it is shown in Fig. 4. Considering just formal medical school teaching, including SSCs and electives, the mean number of hours is 4623, but inclusion of self-regulated teaching takes the mean to 6855 h. On that basis, all 5-year medical courses would appear to be comfortably within the requirement of 5500 h. If however estimated self-regulation learning were not included, then most medical schools would be below 5500 h of formal, timetabled teaching.

We have no data on graduate entry courses, which typically are 4 years in length, but presumably overall teaching time is proportionately less. On the basis of teaching times typical of year 1 and years 3 to 5 (i.e. only one BMS year), and SSCs, but excluding the elective, and proportionately reducing self-regulated learning, mean timetabled teaching time would be about 3450 h. Including self-regulated learning takes the mean total time to about 5350 h, with about half or so of courses vulnerable to falling below the 5500 h. Clearly, there is a need for formal data to be collected from the 4-year graduate entry courses, as our study specifically considered only standard entry 5-year courses.

### The overall pattern of teaching

The big picture of UK medical school teaching is shown in Figs. 2 and 3. It is immediately obvious that the traditional pattern of medical education—basic medical science in the years 1 and 2, followed by clinical studies in years 3, 4, and 5—is still broadly present in UK medical education, at least at the level of timetables. The major basic sciences are taught almost entirely in the first 2 years, at least in a systematic way. It is probable that basic sciences are often referred to and discussed later during clinical teaching, although demonstrating that would need a more detailed, more granular content analysis. Clinical teaching is not restricted solely to years 3, 4, and 5, as it had been previously, as in the GMC’s 1977 survey [52]. Early clinical experience, clinic, and GP sessions are now timetabled within the first 2 years, although they still form only a minor part of the early curriculum. The major thrust of clinical teaching is in clinics, wards, and theatres, with only relatively little dedicated learning of practical skills and little use of simulation. Student-selected components are present in all medical schools, although they are far from the one third of the medical course that *Tomorrow’s Doctors* had originally suggested.

### Medical school differences and problem-based learning

Medical schools vary in the durations of different teaching formats and different teaching contents. That variation is clearly shown in the matrix of Fig. 5. Making sense of Fig. 5 is not easy, but Fig. 7a, b helps, with Fig. 7b being particularly useful as it maps the 25 UK medical schools; the closer the schools are together, the more similar their teaching approach. The first dimension is clearly related to PBL teaching, and the second seems to reflect variation in how structured or unstructured the medical courses are, although these two factors seem to correlate with many other features of the courses (see Fig. 7a).

PBL has been the most controversial and one of the most interesting changes in UK medical education [55]. Understanding this change and the implications remains difficult. Figure 6 shows that PBL schools differ from non-PBL schools on several measures of teaching time. Unsurprisingly, PBL schools have more PBL teaching. PBL schools also have more GP teaching and GP sessions, as well as more ‘unspecified content’. PBL schools have fewer lectures, less specific time on basic medical sciences, and less specified time on the teaching of surgery. Although PBL schools have less timetabled basic medical science teaching, it does not necessarily imply students are exposed to fewer hours of such teaching, as it may occur within specifically timetabled PBL sessions, or in the much larger duration of ‘unspecified content’ which characterises PBL schools. Answers to critical questions about ‘the detailed basic science content of PBL sciences’ [25, 26] will require a different form of data collection involving analysis of specific content within teaching. Figure 7b also demonstrates the uniqueness in the philosophy and approach of PBL schools, with the 10 PBL schools clustered to the left of the plot. It must be noted, though, that there is a clear continuum of PBL [21–23] and non-PBL schools, with variation within the PBL schools as well as variation within the non-PBL schools on the traditional-PBL dimension.

The key questions for PBL (and indeed for any variations in medical school teaching) concern professional outcomes during training and practice. Cavenagh, in comparing traditional and ‘new’ (i.e. mostly problem-based learning) curricula, put it forcefully:

‘The big question … is how successful has the new curriculum been [ … ]. [O] ur first concern must be that doctors are clinically competent, practise evidence-based medicine and are safe practitioners. … If this can be delivered within the context of a supportive educational and clinical environment, where medical students are nurtured in a way that feeds their own humanity and encourages their thirst for learning and knowledge, then with effective recruitment strategies a revised curriculum should achieve the aspirations outlined for *Tomorrow’s Doctors*’ [24](p. 21).

Assessing the extent to which those latter aims have been met is far from straightforward, not least because of the range of the outcome measures required. A ‘rigorous comparison’ [25] of PBL and non-PBL courses will require a wide range of outcome measures, and a start on that will be provided in the *MedDifs* study [1].

### Timetables and actual student behaviour

This study is about timetables, and timetables should, of course, apply to all students in equal ways. Timetables though are an idealisation of an intended curriculum in the minds of those planning and running a medical school. Timetables are also for an idealised student, actual teaching provided varying due to particular placements at different hospitals or GP practices, etc. How timetables relate to what students actually do is a different matter. In a very rare study using detailed diaries of clinical students on rotations, Worley et al. [61] showed that although timetabled hours of lecture teaching were 3–4 h per week, actual student-recorded hours averaged 3 h 12 min a week, with a range from 1 h 11 min to 8 h 24 min. Other forms of teaching showed similar variation across students, with tutorials having a mean of 7 h 54 min with a range of 4 h 12 min to 14 h 7 min and individual study having a mean of 26 h 33 min and a range of 10 h 25 min to 49 h 23 min. Timetables can therefore only say so much about what students are *actually* doing, and mainly are describing what they *should* be doing. Nevertheless, if little actual time is timetabled for an activity, then it is probably a reasonable assumption that little is actually being done on that activity. A corollary is that only a small proportion of notional clinical teaching time on wards may actually be spent on teaching itself [62]. There is also the probability that much real teaching is informal, particularly between student and student, while in hospitals, but also while socialising outside of formal medical education, or anywhere where students chat about the cases they have seen and their interpretation. Such teaching and learning may well be mediated via the social networks that inevitably are developed during medical school [63]. The present study does show different approaches in different medical schools to what *should* be taught, reflecting the different educational philosophies and priorities of the schools. Further studies are needed to address the question of how students within medical schools differ in the *actual* teaching that they receive (and ‘time-logging’ may help [51]). A yet further problem is to assess what of that actual received teaching is *influential and effective* (rather than being perceived as boring, uninteresting, or irrelevant), and perhaps influences subsequent clinical practice or career choices.

### Clarification

We have been asked to make clear, to avoid any possible doubt, that neither this nor the *MedDifs* paper is stating or implying that any of the schools detailed are providing a sub-standard education or are otherwise badly run.

## Conclusions

UK medical schools differ in the format and the content of their teaching, which can be assessed from timetables. Inclusion of the data from Fig. 5 in the UK Medical Education Database (UKMED [64]) will allow other researchers to investigate medical school differences more deeply. Two main patterns underlie the differences, with schools varying in the extent to which they are traditional or PBL-oriented, and the extent to which teaching is structured or unstructured. PBL schools differ in a number of different ways from non-PBL schools, although there are also many broad similarities. The present approach provides a basis both for assessing how teaching changes within UK medical education and also for determining the extent to which teaching differences result in outcome differences later in medical careers.

## Supplementary information

**Additional file 1: Supplementary file 1.** Data for Fig. 5 as Excel file.

## Data Availability

The authors declare that the key aggregated data supporting the findings of this study are available within the article (Fig. 5). Other raw data that support the findings of this study are available from the lead author (Oliver Devine) upon reasonable request and with the agreement of Msico (msico.org).

## Footnotes

1 The Freedom of Information Act was used because in a previous study [38], despite medical schools having voluntarily provided information on assessment, after publication, we had extended problems due to an anonymous medical school suggesting to the editor that we did not have permission to reproduce material and that some details were incorrect. The journal in which that paper was published offered a right of reply to the medical school, but no subsequent submission has ever been received by the journal. By providing information under FoI, the medical schools explicitly put information into the public domain. We also note that in the study of economics teaching, only a minority of universities, seven out of sixteen, would co-operate voluntarily, suggesting that voluntary response from universities cannot in general be relied upon. Likewise, in a study of medical students, only 20 of 33 medical schools gave consent for students to be contacted [39].

2 PBL and CBL are similar but conceptually distinct with an important distinction between them: problem-based learning uses an ‘open enquiry’-based learning method whilst CBL uses a guided enquiry-based learning method [46]. Too few schools in the UK used CBL to make it possible to compare it with PBL and non-PBL, and therefore, it has been included within the PBL group.
